# Supplementary material for: Impact of integrating objective structured clinical examination into academic student assessment: Large-scale experience in a French medical school
Source: PLoS One. 2021 Jan 14;16(1):e0245439. doi: 10.1371/journal.pone.0245439 (PMC7808634; doi:10.1371/journal.pone.0245439)
Supplement: S1 Data — (DOCX) [file pone.0245439.s002.docx]

**S1 Data.** OSCE #1 Script and evaluation grid

**Instructions to the students**

You are on duty night in the emergency room department on June 1, 2019. Your patient, age 43 years old, has presented with chest pain. The report of the initial nurse evaluation and examinations is provided below.

You should perform a detailed examination explaining clearly what you expect from each exam. For example, if you hit the lumbar fossa, you should explain that you are looking for renal pain. The examiner will provide answers to your questions. For example, he will tell you if the renal pain is present or not.

Also, you can ask for results of examinations performed immediately in the emergency room department. The examiner will give you the results of these examinations when available.

After 6 minutes, the examiner will stop the examination procedure and will ask you for your diagnostic hypothesis and the results of related investigations.

**Initial nurse evaluation and examinations:**

Date: June 1^st^, 2019, 6:13am

Patient: Ms. Roussel or Mr. Martin, date of birth 25/03/1976

Reason for medical examination: chest pain that began during the night

Weight 60 kg (female) or 81 kg (male), height 1 m 73 (female) or 1 m 83 (male)

Heart rate: 105/min, SAP/DAP, 105/36 mm Hg

Breathing rate: 16/min; pulsed oxygen saturation: 93% in-room air

Available investigations (to be provided to the student upon request)

Electrocardiogram: sinus tachycardia, right bundle branch block

Urine dip: leucocyte: negative; nitrite: negative; protein: traces; blood: negative; glucose: negative; ketones: negative

Rapid streptococcus test: negative

Chest x-ray: normal

Box examination: n°4

**Grid of evaluation for OSCE #1 (/20):**

|  | **Points (/20)** |
| --- | --- |
| **Behavior-oriented items** | **/2 points** |
| - Hands washed with appropriate hydro alcoholic solution - Proper introduction | 1  1 |
| **Competence-oriented items** | **/18 points** |
| Medical questioning, look for   - Tobacco consumption - Cardiovascular risk factors (diabetes or arterial hypertension or hypercholesterolemia, reason given) - Recognized thrombosis risk factors or previous history of thrombosis (history of thrombosis, cancer, lower limb trauma) | /5 points  1  1  2 |
| If the patient is a male, evaluation of cardiovascular risk factors | 1 |
| If the patient is a female, evaluation of   - history of miscarriage - use of oral contraception | 0.5  0.5 |
| Clinical examination   - Look for right signs of cardiac failure   (hepato jugular reflux, jugular distension, liver pain)   - Perform a cardiopulmonary auscultation - Examination of calves (signs of pain or edema) - Look for Homans sign while explaining how it is done. Spelling the name of Homans but not performing the evaluation is not sufficient     (Penalty if the pain of calves was not found) | /4 points  1  1  1  1  (-1) |
| Give the proper complete diagnosis   - Pulmonary embolism - Associated with left leg deep vein thrombosis - With high clinical probability   (Score zero on examination if the diagnosis of pulmonary embolism is missing) | /6 points  3  2  1 |
| Suggest immediate appropriate investigations   - Electrocardiogram to eliminate an acute coronary syndrome (give the point if asked for at any time during the exam period) - Chest angiography computed tomography (state the necessary precautions before such investigations are performed) | /3 points  1  2 |
| (Do not take into account chest angiography computed tomography if the diagnosis of pulmonary embolism was not made) |  |
| Free written feedback from the examiner   - Empathy - Ability to interview the patient - Medical reasoning |  |
