# Supplementary material for: Impact of integrating objective structured clinical examination into academic student assessment: Large-scale experience in a French medical school
Source: PLoS One. 2021 Jan 14;16(1):e0245439. doi: 10.1371/journal.pone.0245439 (PMC7808634; doi:10.1371/journal.pone.0245439)
Supplement: S2 Data — (DOCX) [file pone.0245439.s003.docx]

**S2 Data.** OSCE #2 Script and evaluation grid

**Instructions to the students:**

You have replaced your co-resident in the cardiology department. The patient is Dominique DUPONT, born on 01/02/1965 (aged 54 years old). You must give the discharge papers to the patient, who has presented with an extensive anterior myocardial infarction, managed with coronary angioplasty and insertion of an active coronary stent. The patient has been hospitalized for 10 days in the cardiology department.

The patient has been smoking 1 pack of cigarettes a day for 30 years prior to hospitalization, had no known medical history, but had not consulted any doctor for the past 5 years. His body mass index is 31 kg/m². Arterial pressure measurements were above 140/90 mmHg during the entire stay. Fasting LDL-cholesterol was measured at 5 mmol/L (2 g/L), fasting glucose at 5.5 mmol/L (1 g/L).

**Instructions:**

You are asked to interview Dominique DUPONT, and explain:

- his condition and the potential complications
- the risk factors and their means of prevention
- the planned follow-up

You are not asked to discuss treatments or medications.

You are not asked to perform a physical exam.

You are not asked to write a prescription.

You will be evaluated on your attitude and the relevance of the information transmitted.

**Grid of evaluation for OSCE #2 (/20):**

| **Item** | **Points (/20)** |
| --- | --- |
| **Behavior-oriented items** | **/6 points** |
| Greets the patient at the beginning and introduces him/herself | 0.5 |
| Ensures that the patient has a good understanding of the information | 0.5 |
| Does not use overly technical language | 0.5 |
| Communication tools: use of active listening, open questions, reformulation | 0.5 |
| Offers help with smoking cessation | 2 |
| Suggests dietary measures (i.e., Mediterranean diet) | 1 |
| Asks whether the patient has a sedentary lifestyle | 1 |
| **Competence-oriented items** | **/14 points** |
| States that myocardial infarction is a serious illness with risks of sequelae and mortality | 2 |
| States that recurrences of myocardial infarction are possible | 2 |
| Cites at least one symptom of recurrence | 0.5 |
| Cites at least one possible late complication | 0.5 |
| Explains that quitting smoking is essential | 2 |
| Explains the need to control hypertension | 2 |
| Explains the importance of cholesterol level control | 2 |
| Explain the importance of weight loss | 1 |
| Explain the importance of regular physical activity | 1 |
| States the importance of regular general medical follow-up | 1 |
| States the importance of regular cardiological follow-up | 1 |
