# Supplementary material for: Impact of integrating objective structured clinical examination into academic student assessment: Large-scale experience in a French medical school
Source: PLoS One. 2021 Jan 14;16(1):e0245439. doi: 10.1371/journal.pone.0245439 (PMC7808634; doi:10.1371/journal.pone.0245439)
Supplement: S3 Data — (DOCX) [file pone.0245439.s004.docx]

**S3 Data.** OSCE #3 Script and evaluation grid

**Instructions to the students:**

You are a medical student at the general surgery department of your university hospital and a nurse asks you to go and see a patient, Mr./Mrs. Dupont, 55 years old, admitted the day before a planned cholecystectomy indicated for the treatment of gallstone disease. The patient is particularly stressed before the intervention and has a few questions for doctors:

He/she underwent an angioedema after taking amoxicillin (which the general practitioner prescribed for treatment of a chronic cough). This angioedema was treated in emergency and he/she was then hospitalized for 2 days of surveillance. The patient is very worried about the cholecystectomy and asks the student to reassure him/her.

The other point is that this intervention is stressful and the patient wonders if it is useful. He/she understands very well the technical part of the intervention but ask for its actual benefit and relevance, since he/she does not feel abdominal pain anymore.

You will be evaluated on the relevance of the answers given and on your general attitude regarding the stress level of the patient.

**Grid of evaluation for OSCE #3 (/20):**

| **Item** | **Points (/20)** |
| --- | --- |
| **Behavior-oriented items** | **/14 points** |
| The student introduces him/herself properly:   - States his/her name - States his/her function | 1  1 |
| The student reassures the patient concerning this allergy, saying that it will be taken into account and that he/she will not receive any penicillin during the hospitalization | 2 |
| The student is empathetic and listens to the patient | 2 |
| The student avoids medical jargon or takes the time to explain it | 2 |
| The student asks the patient if explanations given were understood | 2 |
| The student asks the patient if he/she has any other questions | 2 |
| General attitude (posture, smile, look at the patient in the eyes) | 2 |
| **Competence-oriented items** | **/6 points** |
| The student states that he/she will verify that the medical history of allergy has been mentioned to the doctors in charge (surgeon, anesthesist, and/or anesthesia file) | 2 |
| The student ensures that the patient has no other allergies | 2 |
| The student explains that main aims of the intervention are to avoid:  - recurrences of cholelithiasis  - and potential acute infectious complications | 1  1 |
